# The Integration of Metabolomic and Proteomic Analyses Revealed Alterations in Inflammatory-Related Protein Metabolites in Endothelial Progenitor Cells Subjected to Oscillatory Shear Stress

**DOI:** 10.3389/fphys.2022.825966

**Published:** 2022-02-16

**Authors:** Jie Yu, Jie Fu, Xiaoyun Zhang, Xiaodong Cui, Min Cheng

**Affiliations:** School of Basic Medicine Sciences, Weifang Medical University, Weifang, China

**Keywords:** oscillatory shear stress, endothelial progenitor cells, atherosclerosis, inflammation, metabolomics, proteomics, cardiovascular disease (CVD)

## Abstract

**Background:**

Endothelial progenitor cells (EPCs) play essential roles in vascular repair. Our previous study suggests OSS would lead EPCs transdifferention into the mesenchymal cell that aggravates pathological vascular remodeling. The primary purpose of this study was to apply OSS *in vitro* in EPCs and then explore proteins, metabolites, and the protein-metabolite network of EPCs.

**Methods:**

Endothelial progenitor cells were kept in static or treated with OSS. For OSS treatment, the Flexcell STR-4000 parallel plate flow system was used to simulate OSS for 12 h. Subsequently, an untargeted metabolomic LC/MS analysis and a TMT-labeled quantitative proteomic analysis were performed.

**Results:**

A total of 4,699 differentially expressed proteins (DEPs) were identified, among which 73 differentially expressed proteins were potentially meaningful (*P* < 0.05), with 66 upregulated and 7 downregulated expressions. There were 5,664 differential metabolites (DEMs), of which 401 DEMs with biologically potential marker significance (VIP > 1, *P* < 0.05), of which 137 were upregulated and 264 were downregulated. The Prison correlation analysis of DEPs and DEMs was performed, and the combined DEPs–DEMs pathway analyses of the KGLM database show 39 pathways. Among the DEPs, including the Phosphoserine phosphatase (PSPH), Prostaglandin E synthase 3 (PTGES3), Glutamate–cysteine ligase regulatory subunit (GCLM), Transaldolase (TALDO1), Isocitrate dehydrogenase 1 (IDH1) and Glutathione S-transferase omega-1 (GSTO1), which are significantly enriched in the citric acid cycle (TCA cycle) and fatty acid metabolic pathways, promoting glycolysis and upregulation of fatty acid synthesis. Moreover, we screened the 6 DEPs with the highest correlation with DEMs for predicting the onset of early AS and performed qPCR to validate them.

**Conclusion:**

The comprehensive analysis reveals the following main changes in EPCs after the OSS treatment: dysregulation of glutamate and glycine metabolism and their transport/catabolic related proteins. Disorders of fatty acid and glycerophospholipid metabolism accompanied by alterations in the corresponding metabolic enzymes. Elevated expression of glucose metabolism.

## Introduction

The occurrence and mortality of cardiovascular disease (CVD) are always high, endangering public health and safety. Atherosclerosis (AS) is the main cause of coronary heart disease, cerebral infarction and peripheral vascular disease (Roy et al., 2021). Chronic progressive inflammatory, metabolic diseases caused by the accumulation of lipids from the intima leads to the formation of plaques, which are prone to form in the opening, bifurcation, and bending of an artery. This formation is closely related to the anatomy and hemodynamic characteristics of these portions of vessels (Cheng et al., 2006), which indicates shear stress (SS) plays an essential role in the development of these diseases.

Shear stress is divided into high shear stress (HSS) and low shear stress (LSS), even oscillatory shear stress (OSS). According to previous findings, with the blood flow pattern shifts toward turbulence, OSS (± 4 dynes/cm^2,^ 1Hz) at a bifurcation or bend in a blood vessel can cause endothelial dysfunction, and leads to increased monocyte adhesion, proliferation, and apoptosis, resulting in pro-inflammatory endothelial/endothelial progenitor-mesenchymal transition (Gao et al., 2020; Theofilis et al., 2021; Zhao et al., 2021). However, the mechanism of OSS on atherosclerosis has not yet been fully elucidated (Warboys et al., 2011).

Endothelial progenitor cells (EPCs) are precursors of vascular ECs and can be isolated from bone marrow cord, blood or peripheral blood, from pulmonary artery endothelium or placenta, or from induced pluripotent stem cells. Their main biomarkers are CD34, CD133, and vascular endothelial growth factor (VEGF) receptor 2. There are two type EPCs, namely early EPCs and late-stage EPCs. Early EPCs have the hematopoietic origin and promote angiogenesis through paracrine mechanisms but are unable to produce mature endothelial cells. Late-stage EPCs have the ability to generate mature endothelial cells and then promote angiogenesis *in vitro* and *in vivo* (Peyter et al., 2021). With mobilization by tissue ischemia, cytokine stimulation, or HMG-CoA reductase inhibitors, EPCs can migrate and adhere to endothelial injury and ischemic sites, where they repair and maintain the integrity of vascular endothelial tissue (Tesfamariam, 2016; Kutikhin et al., 2018; Morrone et al., 2021). However, with the presence of cardiovascular risk factors, the number and function of EPCs decreases proportionally (Hur et al., 2004).

It is generally accepted that the shear stress system can simulate the force to vascular endothelial cells (VECs) generated by fluid flow in the vascular lumen. In this study, we treated EPCs with Flexcell STR-4000 parallel plate flow system and then proteomic and metabolomic analyses were performed. A quantitative proteomic analysis based on tandem mass labels (TMTs) was combined with a metabolomic analysis based on untargeted LC–MS to study changes in EPCs in response to OSS (± 4 dyne/cm^2^, 1 Hz). Quantitative reverse transcription (qRT)-polymerase chain reaction (PCR) was performed to validate the proteomic and metabolomic analyses results. Our study could provide novel data for understanding the development of AS.

## Materials and Methods

### Cell Culture and Experimental Groups

Endothelial progenitor cells were extracted from human umbilical venous blood and cultured *in vitro.* Briefly, mononuclear cell were getted from human peripheral blood by density gradient centrifugation and cultured with EGM-2 MV (Lonza,Sweden) complete medium (Bei et al., 2017). Generations 5–7 of EPCs grown in the logarithmic phase were used. EPCs were randomly divided into two groups: static group (static), in which EPCs were kept in static for 12 h, and OSS group, in which OSS of ± 4 dynes/cm^2^ at 1 Hz was applied on EPCs for 12 h. The OSS is applied by the Flexcell STR-4000 parallel plate flow system through a computer-controlled mechanical loading system (Streamer). The EPCs were seeded on glass slides coated with Fibronectin (Fn, Sigma, United States) at a concentration of 0.5 μg/cm^2^. The glass slides with EPCs were carefully placed in the Streamer, and OSS was applied. This study was approved by the Ethics Committees Review Board at Weifang Medical University, Weifang, China.

### Sample Preparation

After treatment, the glass slides with EPCs were removed from the Flexcell STR-4000 system, placed in a sterile four-well plate, washed once with 1× PBS, and then, the PBS was removed as cleanly as possible. Metabolomic samples (two groups with 16 samples) were prepared as follows: To quench cell growth, liquid nitrogen was applied to the bottom of the outer walls of the four-well plate containing the cell slides for 10 s. Methanol and distilled aqueous solution were prepared at a ratio of 4:1 by volume. Then, a total 1 mL of this methanol-water solution was added twice. The cells were scraped from the slides with a disposable cell scraper and transferred to a clean and sterile 1.5 ml centrifuge tube. The cells were stored at −80°C. Proteomic samples (two groups with six samples) were prepared as follows: at least 1 × 10^7^ EPCs were used in each group of samples. The cells were digested with 0.25% trypsin. The cell suspension was prepared with EGM-2MV complete medium, centrifuged at 1,000 rpm for 5 min at room temperature. EPCs suspended with 1×PBS, and then centrifuged at 1,000 rpm for 5 min at room temperature. This process was repeated 3 times. The cells were stored at −80°C, and transported in dry ice to Shanghai Ouyi Biotechnology Co., Ltd., for testing.

### Untargeted Metabolomic LC/MS Analysis

For the metabolic experiment, a liquid mass spectrometry system (Thermo Fisher Scientific) consisting of a Dionex U3000 UHPLC ultrahigh-performance liquid phase tandem QE plus a high-resolution mass spectrometer was used for LC–MS/MS analysis. Chromatographic separation was performed using an ACQUITY UHPLC T3 column (100 × 2.1 mm; 1.8 μm, Waters). Mobile phase A was composed of 0.1% formic acid in the water, and mobile phase B was composed of acetonitrile containing 0.1% formic acid. The loading volume in positive and negative ESI mode was 2 μl. The elution gradient settings were as follows: 5% B (0–2 min), 5–25% B (2–4 min), 25–50% B (4–8 min), 50–80% B (8–10 min), 80–100% B (14–15 min), 100% to 5% B (15–15.1 min), and 5% B (15.1–16 min); the flow rate was 0.35 mL/min. The mass spectrometry conditions were a spray voltage (V) of 3800/−3000; a capillary temperature (°C) of 320/320; an auxiliary (Aux) gas heater temperature (°C) of 350/350; a sheath gas flow rate (Arb) of 35/35; an aux gas flow rate (Arb) of 8/8; an S-lens RF level of 50/50; a mass range (m/z) of 100--1000/100--1000; a mass range (m/z) of 70000/70000; MS/MS resolution of 17500/17500; an NCE and stepped NCE of 10, 20, and 40 and of 10, 20, and 40, respectively. UNIFI 1.8.1. the software was used for raw data acquisition, and raw data were processed with the metabolomics processing software Progenesis Q.I. v2.3 (Non-linear Dynamics, Newcastle, United Kingdom). Then, the specific names and biological functions of differentially expressed proteins and metabolites were identified through the UniProt^1^, KEGG^2^, and KGLM^3^ databases, and enrichment analyses were performed.

### Tandem Mass Labels-Labeled Quantitative Proteomic Analysis

For the proteomic analysis, chromatographic separation was performed with high-pH separation liquid chromatography, and Q Exactive and EASY-nLC 1200 systems were used for the LC-MS/MS analysis and data analysis. Reversed-phase chromatography separation was performed with an Agilent 1100 HPLC system as follows: An Agilent Zorbax Extend-C18 narrow bore column (2.1 × 150 mm, 5 μm) was used, and the detection wavelengths were UV 210 nm and 280 nm. Mobile phase A consisted of ACN-H_2_O (2:98, v/v), and mobile phase B consisted of ACN-H_2_O (90:10, v/v). The gradient elution conditions were 98% A (0∼8 min); 98∼95% A (8∼8.01 min); 95∼75% A (8.01∼48 min); 75∼60% A (48∼60 min); 60∼10% A (60∼60.01 min); 10% A (60.01∼70 min);10∼98% A (70∼70.01 min); and 98% A (70.01∼75 min). The flow rate was 0.300 mL/min. The chromatography conditions were as follows: a sample was loaded onto an Acclaim PepMap 100, which is a 100 μm × 2 cm precolumn (RP-C18, Thermo Fisher Scientific), at a 300 nL/min flow rate, and then, the sample was separated on an Acclaim PepMap RSLC analytical column (75 μm × 15 cm, RP-C18, Thermo Fisher Scientific). Mobile phase A was composed of H_2_O-FA (99.9:0.1, v/v); mobile phase B was composed of ACN:H_2_O: FA (80:19.9:0.1, v/v/v). The gradient elution conditions were 5–30% B (0∼40 min); 30–50% B (40∼54 min); 50–100% B (54∼55 min); and 100% B (55∼60 min). The conditions for mass spectrometry were as follows: The MS mass resolution was 70,000; the automatic gain control value was 1e6; the maximum injection time was 50 ms; the m/z range consisted of a charge-to-mass ratio of 300-to-1600; the collision energy was 32; the MS/MS resolution was 17,500; the automatic gain control was 2e5, the maximum ion injection time was 80 ms, and the dynamic rejection time was 30 s. Protein-metabolite interaction networks provide visible interactions between functionally relevant differentially expressed proteins (DEPs) and differentially expressed metabolites (DEMs). This experiment uses the Pearson correlation calculation method to calculate DEPs-DEMs correlations, allowing proteomics and metabolomics data to be integrated to obtain common pathways and interactions between DEPs and DEMs.

### Real-Time Fluorescence Quantitative PCR

Total RNA was extracted by fully lysing cells on ice with TRIzol reagent (Thermo-Invitrogen Ambion, United States). Then, total RNA was reverse transcribed to cDNA using Evo M-MLV RT Premix for qPCR (AG, China) reverse transcription kit according to the instructions. The qPCR was performed according to the instructions of SYBR Green Premix Pro Taq HS qPCR Kit (AG, China), and the reaction conditions of QuanStudio fluorescent quantitative PCR instrument (Thermo Fisher Scientific, United States). The reaction conditions were set to 95°C, 5 s, 60°C, 30 s for 40 cycles. The amplification efficiencies of GSTO1, IDH1, TALDO1, GCLM, PSPH, and PTGES3 were calculated statistically according to Equation 2^–ΔΔ*CT*^, using GAPDH as an internal reference. The primer sequences for these genes are listed in Table 1:

**TABLE 1:** The primer sequences in qPCR.

Gene name		Primer sequences
GSTO1	Forward	AGCTAGAGGAGGTTCTGACTAA,
	Reverse	TTTAACTTCATTGCTTCCAGCC;
IDH1	Forward	GATGGCAAGACAGTAGAAGCAGAGG
	Reverse	ATGGAAGCAATGGGATTGGTGGAC
TALDO1	Forward	TCAGCAAGGACCGAATTCTTAT
	Reverse	TCAGCAAGGACCGAATTCTTAT
GCLM	Forward	GGGCACAGGTAAAACCAAATAG
	Reverse	TTTTCACAATGACCGAATACCG
PSPH	Forward	GGAAGCTTTTCTACTCAGCAGA
	Reverse	CACAGATTTTGGCTAGCTCATC
PTGES3	Forward	GTTGTCTCGGAGGAAGTGATAA
	Reverse	CTTTTGTTAACCTTGGCCATGA
GAPDH	Forward	GTCATCAATGGAAATCCCATCA
	Reverse	CCAGTGGACTCCACGACGTAC

## Results

### Protein Identification and Classification After Oscillatory Shear Stress Stimulation

**FIGURE 1:**
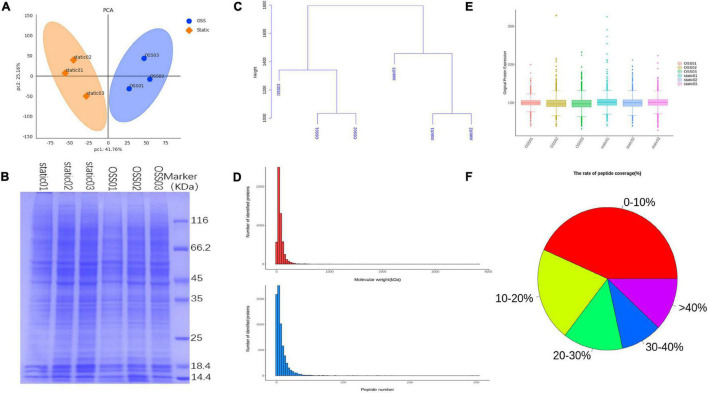
Diagram showing the analysis of 6 samples in the OSS (*n* = 3) and static (*n* = 3) groups using a Flexcell STR-4000 parallel plate flow system. **(A)** Principal component analysis (PCA) showing the credible protein expression in the OSS and static groups. **(B)** SDS- polyacrylamide gel electrophoresis (SDS–PAGE) electropherogram showing the OSS and static proteins. **(C)** Hierarchical clustering dendrogram showing the Euclidean distances of the OSS and static samples. **(D)** The number of proteins corresponding to different molecular weights of OSS and static group bands and the distribution of the number of peptides corresponding to each qualitatively assessed protein. **(E)** Box plots show credible protein expression in the OSS and static groups. **(F)** Comparison of each peptide in OSS and static groups against background databases and the coverage index map showing the peptides relative to complete protein sequences.

To compare the protein profiles at static and OSS group, TMT 6-plex labels were used for each sample. And then the relative abundance of DEPs were determined. The result showed that protein expression was significant differences between the OSS (*n* = 3) and static (*n* = 3) group. A principal component analysis (PCA) showed that the overall difference in the protein profile between the groups was obvious, and the protein analysis for each group was reproducible (Figure 1A). SDS-polyacrylamide gel electrophoresis (SDS-PAGE) revealed that the protein bands were evenly distributed (Figure 1B). The hierarchical clustering dendrogram showing the sample Euclidean distance based on credible expression quantitative data also indicated that the protein characteristics between the static and OSS groups were different (Figure 1C).

The distribution of the proteins with different molecular masses and the peptide number of each qualitative protein in the raw data was uniform and consistent (Figure 1D). The box-plot showed small fluctuations in the credible protein expression levels obtained through an analysis of the static and OSS group (Figure 1E). Database search software was used to compare each peptide with the background database. The coverage index of the peptide relative to the complete protein sequence was obtained (Figure 1F).

### The Metabolic Pathways of Differentially Expressed Proteins Involve 13 Biological Functions Associated With the Development of Inflammation

**FIGURE 2:**
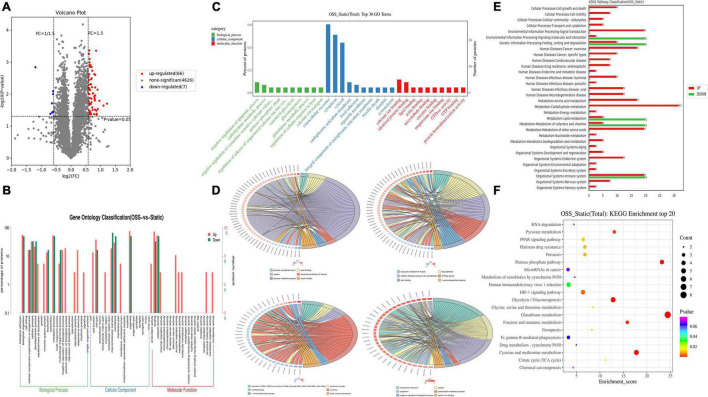
DEPs between OSS and STATIC groups and the pathways involved were analyzed. **(A)** DEPs volcano plot showing OSS and static proteins. There were 73 DEPs. Red indicates upregulated DEPs, and blue indicates downregulated DEPs (fold-change = > 1.5 and *p*-value < 0.05). **(B)** Comparison of DEPs and protein species distribution in the GO Level 2 figure. **(C)** GO enrichment analysis showing the 30 most enriched proteins (the GO terms correspond to the number of DEPs greater than 1 in the three GO classifications, and 10 terms with the greatest to least enrichment are shown according to the corresponding –log10P value of each protein). **(D)** GO enrichment analysis chord diagram (the number of differentially expressed proteins in each comparison group greater than 3 and less than 50 is shown, with the first 6 items shown in descending order of the –log10 *P*-value corresponding to each term), showing the relationship between the selected GO term and the corresponding DEPs in the list. **(E)** Upregulated-DEPs and downregulated-DEPs in the KEGG Level 2 level distribution diagram. **(F)** Bubble chart showing the KEGG enrichment analysis with the 20 most enriched proteins (sorted according to the corresponding –log10P value of each term in descending order).

In the proteomic dynamics experiment, 4,699 DEPs were found between the static and OSS groups. A total of 73 differentially expressed proteins were identified on the basis of a fold change > = 1.5 and a *P* value < 0.05 as criteria. We found that the expression of 66 of these DEPs was upregulated and 7 of these DEPs was downregulated (Figure 2A). According to GO and KEGG analyses (Figures 2B,F), these proteins were mainly enriched in 13 functional categories. These DEPs were mainly involved in muscles, carbohydrate metabolism, myelin, local adhesion, protein binding, GTPase activity, amino acid metabolism, RNAs, and small subunits. Basal protrusions, exosome release, carbohydrate metabolism, negative regulation of apoptosis, and calcium ion binding were also enriched with these differentially expressed proteins (Figures 2B–D). Among these categories, glycometabolism, amino acid metabolism, fatty acid metabolic, energy metabolism, and hypoxia-inducible factor-1 signaling (Figures 2E,F) were enriched with the most differentially expressed proteins between static and OSS group. Notably, all these pathways are closely associated with the development of inflammation. These pathways might enhance metabolic effects after OSS treatment in EPCs, contributing to the development of AS.

### Differential Metabolites Reflect Changes in L-Glutamate, Beta-D-Glucose, Spermine, Ornithine, Choline, and Indicate More Inflammation-Related Metabolic Markers

**FIGURE 3:**
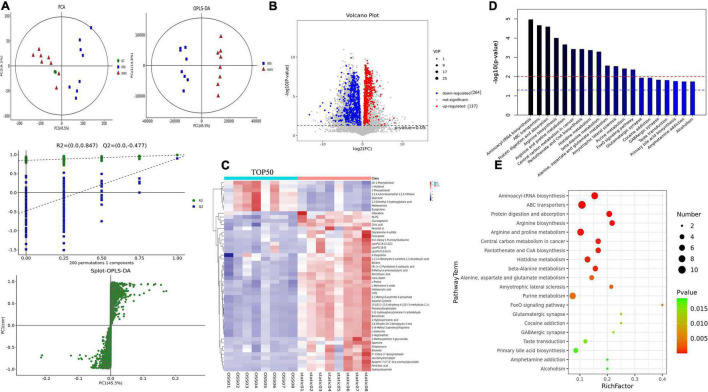
Diagram showing a total of OSS (*n* = 8) and static (*n* = 8) samples analyzed with a Flexcell STR-4000 parallel plate flow system. **(A)** OSS and static PCA, partial least squares analysis (PLS-DA), orthogonal partial least squares analysis (OPLS-DA), and response permutation testing (RPT) diagrams. **(B)** For OSS and static DEMs volcano diagrams, 401 DEMs are shown, with red indicating DEMs upregulation and blue indicating DEMs downregulation (VIP > 1 and *p*-value < 0.05). **(C)** Hierarchical clustering analysis of the expression levels of all significantly DEMs and the 50 most DEMs based on the VIP value. Color changes from blue to red indicate that the change in the expression of metabolites from low to high. **(D)** The *p*-value in a metabolic pathway indicates the significance of the protein enrichment of the metabolic pathway. The red line indicates that a *p*-value of 0.01, and the blue line indicates that a *p*-value of 0.05. **(E)** TOP20 metabolic pathway bubble diagram showing that the greater the enrichment factor is, the greater the degree of enrichment and that the greater the number of metabolites is, the greater enrichment in a pathway.

PCA and supervised partial least square analysis (PLS-DA) model showed that the metabolic profiles of for EPCs treated with OSS or kept in static were significantly different. This study was based on 200 response permutation tests (RPTs). With a fixed X matrix, R2 = (0.0,0.847), Q2 = (0.0, 0.477), we found the rightmost R2 and Q2 points at the highest positions, and the Q2 line was significantly lower than the R2 line and was effectively separated. This outcome indicated that the interpretation and prediction of the model verification parameters were effective and that the model was successfully established. In addition, an orthogonal partial least squares–discriminant analysis (OPLS–DA) eliminated noise that was not related to the classification, improved the analytical power and effectiveness of the OSS and static metabolite models, and maximized the difference between the OSS and static groups (Figure 3A).

Variables with a variable importance of projection (VIP) > 1 and *P* < 0.05 were selected as potential biomarkers, and 401 DEMs were identified, of which the level of 137 metabolites was increased, and that of 264 metabolites was decreased. And 111 DEMs were found in the KEGG database associated with specific terms and related biological functions. These DEMs, including L-glutamate, beta-D-glucose, spermine, ornithine, choline, and Betaine, all showed reduced expression after OSS treatment (Figure 3B).

Next, the Top 50 DEMs were identified with the most potential of inflammation-related metabolic markers, and a heatmap analysis was performed (Figure 3C); each colored cell on the heatmap corresponds to the concentration value in the data table. Through KEGG enrichment analysis, it was found that the metabolic pathways were mainly enriched in the following biological functions: aminoacyl biosynthesis, amino acid metabolism, protein transport, and absorption, coenzyme metabolism, the FoxO signaling pathway, central carbon metabolism of cancer, metabolism, and muscles (Figures 3D,E).

### Differentially Expressed Proteins-Differential Metabolites Correlation Analysis Was Performed and Six Differentially Expressed Proteins Were Screened and Verified

**FIGURE 4:**
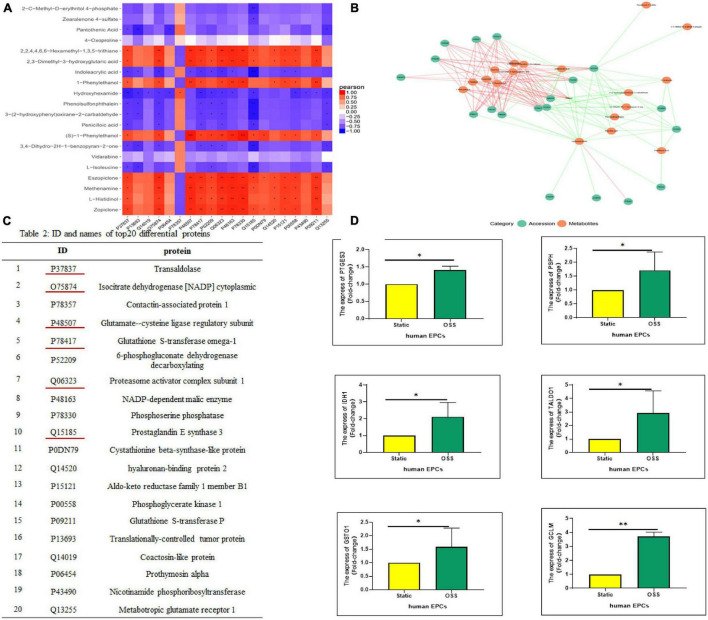
Correlation analysis between DEPs and DEMs. **(A)** A correlation heatmap showing the TOP20 results of the correlation analysis between DEPs and DEMs, orange–red indicates a positive correlation, blue indicates a negative correlation, ^***^p-value < 0.001, ^**^p-value < 0.01, and *p-value < 0.05. **(B)** The correlation between the DEPs and DEMs response intensity data based on a Pearson correlation analysis, with a *p* value < = 0.05 selected to draw a network diagram. Red represents a positive correlation, and the green represents a negative correlation. **(C)** IDs and protein names of the TOP20 DEPs in the UniProt database. **(D)** Real-time polymerase chain reaction (qPCR) shows PSPH, PTGES3, GCLM, TALDO1, IDH1, and GSTO1 expression was significantly elevated after OSS, *n* = 3, *p-value < 0.05, ^**^p-value < 0.01 compared with Static by Mann-Whitney U test.

To better understand the changes in OSS on the biological functions of EPCs, the obtained proteomics and metabolomics data were integrated. The Pearson correlation calculation method was used to evaluate the correlation between DEPs and DEMs, and a correlation heatmap was drawn showing the 20 most highly correlated DEPs and DEMs (TOP 20); there were 170 pairs with *P* < 0.05, 55 pairs with *P* < 0.01 and 3 pairs with *P* < 0.001 (Figure 4A). Moreover, according to the TOP20 results of the correlation analysis between the DEPs and DEMs, the relationship pairs with a *P* < = 0.05 were selected to draw the network diagram. The red line represents a positive correlation. The green line represents a negative correlation, and the thickness of the line represents the value of the correlation coefficient (Figure 4B). Figure 4C lists the specific names of the TOP20 DEPs and the corresponding IDs in UniProt. DEPs with the most prominent correlation were verified by qPCR (Figure 4D). The expression of PSPH, PTGES3, GCLM, TALDO1, IDH1, and GSTO1 increased significantly, which is highly consistent with proteomic analysis.

### The Combined Differentially Expressed Proteins-Differential Metabolites Pathway Significantly Focuses the Glycolytic, Citric Acid Cycle, and Fatty Acid Metabolic Pathways

**FIGURE 5:**
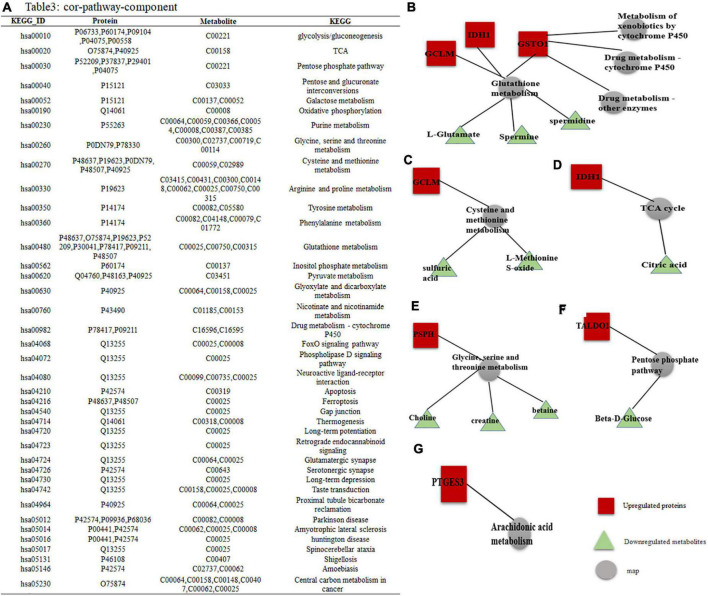
DEPs–DEMs combined pathway analysis. **(A)** Specific KEGG IDs were found to be enriched in 39 metabolic pathways; the specific names of the DEPs and DEMs are shown. **(B–G)** Interaction network of DEPs-DEMs, red indicates upregulated proteins or metabolites, green indicates downregulated proteins or metabolites, and gray represents metabolic pathways.

The enrichment analysis showed that the identified DEPs and DEMs were significantly enriched in specific pathways. A total of 39 metabolic pathways were enriched with differentially expressed molecules, as shown in Figure 5A (*P* < 0.05).

Among them, GO and KEGG analysis of DEPs and DEMs with high enrichment scores yielded a total of 9 metabolic pathways. Combined pathway analysis of the proteins we tracked by DEPs and DEMs showed that these proteins were involved in 6 subnetworks: (1) GCLM, IDH1, and GSTO1 all regulate glutathione (GSH) metabolism, three of which are downregulated including L-glutamate, spermine and spermidine metabolites, while GSTO1 is involved in three metabolic pathways: drug metabolism-cytochrome P450, drug metabolism-other enzymes and metabolism of xenobiotics by cytochrome P450 (Figure 5B); (2) GCLM is involved in the metabolism of cysteine and methionine, of which two metabolite expressions are downregulated including sulfate, methionine sulfoxide (Figure 5C); (3) IDH1 is involved in the TCA cycle to decrease citric acid expression (Figure 5D); (4) PSPH is mainly involved in the metabolism of Glycine, serine and threonine, of which the expression of three metabolites is downregulated including choline, creatine, and Betaine (Figure 5E); (5) TALDO1 is involved in the pentose phosphate pathway to bring down Beta-D-glucose (Figure 5F); and (6) PTGES3 regulates the arachidonic acid metabolic pathway (Figure 5G).

## Discussion

Cardiovascular disease remains one of the leading causes of human mortality (Zhao et al., 2019). Although research on AS, the main type of CVD, has been pervasive, the specific mechanism of AS is still unkown. AS is a chronic progressive inflammatory disease, and metabolic changes play an important role in the pathophysiology of AS. Moreover, the development of AS might be related with OSS. Previous studies have shown that many basic processes in EPCs by affecting the expression and modification of thousands of genes, proteins and metabolites. Therefore, new theories based on systems biology are needed to fully clarify the interaction between mechanical force and EPCs.

A previous study found that monocytes in the body stimulated by inflammation-inducing factors increased carbohydrate metabolism. Glycolysis was found to play a decisive role in promoting inflammation (leading to the progression of AS) (Groh et al., 2018). We found that TALDO1 was highly expressed under OSS intervention, which promoted glucose metabolism and thus contributed to the development of AS. In addition, the two rate-limiting intermediates in the TCA cycle are core eukaryotic energy metabolites that accumulate in the body, further stimulating the development of AS (Liang et al., 2021). And we have demonstrated that the expression of IDH1, a key enzyme of the TCA cycle, is increased after OSS intervention. Some studies have suggested that increased IDH1 and TCA circulating activity in pro-inflammatory cells (Koenis et al., 2018). Also the increased expression of IDH1 and TCA circulating activity could demonstrate inflammatory expression. Studies have shown that changes in amino acid metabolism are closely related to the occurrence of AS. For example, in patients with cardiovascular disease, some branched-chain amino acids (BCAAs) are considered to be early biomarkers of cellular immune activation (Gulasova et al., 2020; Zaric et al., 2020). The differential gene PSPH can directly regulate Glycine (Gly), serine (Ser), and threonine metabolism. And Gly can remodel ECs, reduce the aggregation of macrophages, induce the apoptosis of macrophages, and ameliorate atherosclerosis (Wang et al., 1999).

We found that high GCLM expression after OSS promoted cysteine and methionine metabolism, resulting in increased serum concentrations of Hcy. Moreover, GSH metabolism further contributes to fatty acid metabolism. It is generally believed that the disruption of fatty acid metabolism is a crucial link in the development of AS. Studies have shown that an increase in the concentration of phenylalanine indicates inflammation and impaired immune activation (Gulasova et al., 2020). A study has shown that amino acid metabolism is disordered in AS. A particularly in-depth study into the role of amino acids in stabilizing inflammation revealed that glutamine, which can synthesize GSH, is vital for macrophage-induced IL-1 production in response to LPS stimulation. Glutamine also plays a potential role by feeding the arginine synthesis pathway, facilitating an increase in the microbial capacity of NO macrophages (Wallace and Keast, 1992). At the same time, a study has shown that Glycine plays an obvious role in many biological functions. For example, Glycine can reduce oxidative stress markers and Glycine supplementation can lead to anti-inflammatory effects. Studies have shown that the risk of coronary heart disease is negatively correlated with Glycine concentration in serum (Zaric et al., 2020). In addition, supplementation with the antioxidant GSH can exert an antioxidant effect to protect the vascular endothelium and reduce inflammation (Ruiz-Ramirez et al., 2014). Also, specific metabolites play a crucial role in the AS process. For example, studies have shown that different levels of the metabolite Betaine may inhibit the formation of atherosclerotic plaques by exerting anti-inflammatory effects, thereby inhibiting the occurrence and development of AS (Lv et al., 2009; Wang et al., 2011).

After OSS intervention in EPCs, PSPH expression was upregulated, regulating cysteine and methionine metabolism, eventually leading to a significant decrease in Betaine. A study showed that after cells were subjected to OSS, the betaine level decreased significantly and that 5-hydroxytryptophan, a potential biomarker of DEM with reduced concentrations after experiencing OSS intervention, was incorporated into apolipoprotein A-I, impairing cholesterol efflux activity and high-density lipoprotein biogenesis (Zamanian-Daryoush et al., 2020), promoting the occurrence and development of AS. β-Alanine and L-histidine are known to be involved in the synthesis of carnosine, which can delay inflammation and fight AS (Caruso et al., 2019). However, the expression of both beta-alanine and L-histidine was decreased in the OSS context, and therefore, they can be used as potential biomarkers for the diagnosis of AS to be further studied in depth. Moreover, biliverdin has been proven to be key to regulating cell proliferation, apoptosis and antioxidant defense (Ayer et al., 2016). Data have shown that the biliverdin level was reduced, which promoted the progression of AS. It was found that PTGES3 was highly expressed after OSS action and that PTGES3 could directly regulate arachidonic acid metabolism. Arachidonic acid is known to promote vasoconstriction and platelet agglutination and chemotaxis of neutrophils, further promoting the development of inflammation and AS.

Also, the enrichment of DEPs and DEMs in purine metabolism was more obvious after OSS stimulation. Purine metabolism in the body increases the serum Uric acid (UA) level. A study has shown that UA reduces the availability of nitric oxide (NO) and forms peroxynitrite, which can cause DNA damage and lipid peroxidation, leading to endothelial cell dysfunction and death. Therefore, the blood solubility of UA is positively correlated with the occurrence of AS and is a biomarker of cardiovascular disease risk (Yu and Cheng, 2020).

Moreover, we found that the expression of UA in EPCs was reduced after OSS, and it is possible that the synthesized UA was released into the blood, promoting the development of AS. When attacked by inflammatory factors, the death rates of monocytes, vascular smooth muscle cells (VSMCs) and ECs increase, which further stimulates the occurrence and development of AS (Ackers-Johnson et al., 2015; Paone et al., 2019). For example, a study has shown that the reduced apoptosis of macrophages and increased endocytosis in blood vessel walls was realized by knocking down the lncRNA MAARS, which attenuated the progression of AS (Simion et al., 2020); yam glycosides have also been shown to inhibit oxidative stress, inflammation and apoptosis to reduce postmenopausal AS (Yang et al., 2019). Furthermore, another form of cell death, ferroptosis, is prevalent in AS. Ferroptosis is a newly discovered type of programmed cell death in which the metabolism of lipid oxides in cells and the accumulation of lipid reactive oxygen species leads to redox imbalance in cells (Dolma et al., 2003),and GCLM can promote ferroptosis (Meier et al., 2021). A study has shown that ferroptosis occurs at the beginning and developmental stages of AS. Inhibiting ferroptosis can alleviate AS by reducing lipid peroxidation and endothelial dysfunction of AECs (Bai et al., 2020). Therefore, various mechanisms have been used to inhibit the apoptosis of three central cells of AS, macrophages, ECs, and VSMCs, or to enhance the ability of EPCs and ECs to inhibit the occurrence and development of AS.

After validation, we found that the combination of differential genes PSPH, PTGES3, GCLM, TALDO1, IDH1, and GSTO1 has the ability to act as an early biomarker for the development of AS. Meanwhile, the possibility of delaying the progression of AS by modulating certain metabolic pathways with high enrichment fractions and/or pathway-related metabolites is an element that requires further analysis. For example, directly regulating the purine pathway *in vivo*, controlling the production of UA, and regulating the NOX pathway slows the aging of EPCs and enhances their vascular repair capabilities. Therefore, the *in vitro* expansion and anti-inflammatory ability of EPCs are essential in the treatment of AS. In addition, by increasing the activity of PSPH in the body, the rate of serine production is increased, which regulates the antioxidant capacity and anti-inflammatory capacity of EPCs, thereby delaying the development of AS. Alternatively, the biological functions of EPCs can be adjusted by directly administering relevant small molecules *in vivo* to enhance their proliferation, migration, and adhesion capabilities to fight inflammation and repair vascular damage. For example, direct administration of an appropriate dose of arginine can enhance cell function, delay the development of AS, and reduce the occurrence rate of AS (Cooke et al., 1992). However, this is all fundamental theory, and further in-depth research is needed on how to formulate the therapeutic dosages, the optimal route of administration, and the application to the clinic.

Our study reveals a comprehensive exploration into the mechanism of action of EPCs under the stimulation of OSS through molecular reactions, providing a theoretical basis for the occurrence and development of cardiovascular diseases, related mechanisms, and potential countermeasures. This study only provides a foundation for further study. However, it does not reveal a comprehensive exploration in determining the regulatory mechanism(s) of the pathways enriched with a combination of differentially expressed proteins and differentially expressed metabolites needed for further research.

## Conclusion

A combination of protein analysis, metabolic analysis, and protein metabolism analysis was performed on static EPCs and EPCs after OSS intervention. It is speculated that the inflammatory pathways involve multiple modalities (sugar metabolism, energy metabolism, amino acid metabolism and cell death), potential mechanisms (the aminoacyl biosynthesis pathway, ABC transporters, etc.), and potential treatment methods. This study provides a theoretical basis and essential data for future research.

## Data Availability Statement

The raw data supporting the conclusions of this article will be made available by the authors, without undue reservation.

## Ethics Statement

The Ethics Committee of Weifang Medical University approved the use of human cord blood for this study.

## Author Contributions

XC and MC concept and design. XZ, JY, and JF proteomic and metabolomic analyses and interpretations, statistical analyses, and manuscript drafting. All authors reviewed and edited the manuscript draft and approved the final manuscript.

## Conflict of Interest

The authors declare that the research was conducted in the absence of any commercial or financial relationships that could be construed as a potential conflict of interest.

## Publisher’s Note

All claims expressed in this article are solely those of the authors and do not necessarily represent those of their affiliated organizations, or those of the publisher, the editors and the reviewers. Any product that may be evaluated in this article, or claim that may be made by its manufacturer, is not guaranteed or endorsed by the publisher.

## Footnotes

1
https://www.uniprot.org/

2
https://www.kegg.jp/

3
https://www.kegg.jp/kegg/xml/

## Abbreviations

CVD: cardiovascular disease
AS: atherosclerosis
SS: shear stress
HSS: high shear stress
LSS: low shear stress
OSS: oscillatory shear stress
EPCs: endothelial progenitor cells
VECs: vascular endothelial cells
ECs: endothelial cells
DEPs: differentially expressed proteins
DEMs: differentially expressed metabolites
UA: uric acid
AA: amino acid
BCAAs: branched chain amino acids
Met: methionine
Hcy: homocysteine
Gly: Glycine
Gln: glutamine
GSH: glutathione
Ser: serine
NO: nitric oxide
Hcy: homocysteine
LPS: lipopolysaccharide
IL-1: interleukin-1
VSMCs: vascular smooth muscle cells
NOX: nitrogen oxide
ROS: reactive oxygen species
PCA: principal component analysis
RPT: response permutation testing
PLS-DA: partial least squares–discriminant analysis
OPLS-DA: orthogonal partial least squares–discriminant analysis
VIP: variable importance of projection
SDS-PAGE: SDS-polyacrylamide gel electrophoresis
qPCR: Real-time polymerase chain reaction
PSPH: phosphoserine phosphatase
PTGES3: prostaglandin E synthase
GCLM: glutamate–cysteine ligase regulatory subunit
TALDO1: transaldolase
IDH1: isocitrate dehydrogenase cytoplasmic
GSTO1: glutathione S-transferase omega-1.
